# Breeding Partners Have Dissimilar Foraging Strategies in a Long‐Lived Arctic Seabird

**DOI:** 10.1002/ece3.70816

**Published:** 2025-01-23

**Authors:** Marianne Gousy‐Leblanc, Allison Patterson, H. Grant Gilchrist, Vicki L. Friesen, Kyle H. Elliott

**Affiliations:** ^1^ Department of Natural Resource Sciences McGill University Ste‐Anne‐de‐Bellevue Québec Canada; ^2^ Department of Integrative Biology University of Windsor Windsor Ontario Canada; ^3^ Environment & Climate Change Canada Ottawa Ontario Canada; ^4^ Department of Biology Queen's University Kingston Ontario Canada

**Keywords:** breeding pair similarity, foraging behavior, GPS accelerometers, thick‐billed murre, *Uria lomvia*

## Abstract

For long‐lived species with biparental care, coordination and compatibility in the foraging behavior of breeding mates may be crucial to successfully raise offspring. While high foraging success is clearly important to reproductive success, it might be equally important that the mate has a complementary foraging strategy. We test whether breeding partners have similar or dissimilar foraging strategies in a species where both partners share breeding responsibilities and exhibit high mate fidelity (thick‐billed murre; *Uria lomvia* ). To examine whether thick‐billed murres showed complementary in foraging strategies, we attached GPS accelerometers to both partners within 40 thick‐billed murre chick‐rearing pairs. Individuals within a breeding pair were dissimilar in their foraging trip distance and in their number of dives during foraging trips compared to randomized pairs. Breeding partners were also more similar in wing length than randomized pairs. This result could be related to individual quality as individuals select similar sized partners or select sites that lead to similar sized partners. We conclude that foraging strategy diversity could be maintained in this population either because individuals prefer partners with foraging strategies complementary to their own, or because partners diverge in foraging strategies over multiple breeding season together.

## Graphical abstract

In long‐lived species with biparental care, such as the thick‐billed murre, coordination of foraging strategies between mates may be crucial for raising offspring. This study found that breeding partners had dissimilar foraging behaviors, such as trip distance and dive numbers, compared to randomized pairs, yet they were more similar in wing length. This suggests that foraging strategy diversity may be maintained due to complementary foraging strategies or divergence over time between partners.

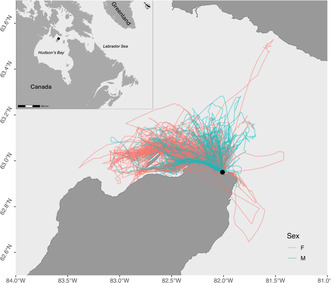

## 1 Introduction

The choice of a breeding partner is a crucial decision for most monogamous species as this choice may have an important effect on reproductive success. In particular, selecting a partner with complementary behaviors may be a particularly important factor for reproductive success. For instance, Montagu's harriers ( *Circus pygargus* ) that pair with a mate dissimilar in personality plasticity have higher reproductive success (Rabdeau et al. 2021); conversely, in the mound‐building mouse ( *Mus spicilegus* ), pairing with an individual of similar personality increases the probability of breeding (Rangassamy et al. 2015).

This relationship is especially meaningful for long‐lived species with biparental‐care, where cooperation amplifies the importance of compatibility due to the costs of sexual conflict: if parental effort in those species differs between mates, one mate's condition could decrease if partners are incompatible, limiting their ability to invest in current or future broods, affecting both partners' long‐term fitness (Royle, Hartley, and Parker 2002; Griffith 2019; McCully et al. 2022). In those species, the combination of phenotypic traits within a breeding pair may be key in pair‐bond formation and cooperation to successfully rear offspring (Holtmann et al. 2021). Individuals should select a partner that will both enhance the performance of the pair as a unit over the entire breeding season (e.g., territorial acquisition and defense, and offspring‐rearing) and also optimize their own fitness (Black 1996; Bried and Jouventin 2002; Griffith 2019).

Compatibility of breeding partners may involve several traits, including morphological features (Leal et al. 2019), behavior (Sommer‐Trembo, Schreier, and Plath 2020), body condition (González‐Medina et al. 2020) and immune function (Sin et al. 2015). Compatibility within breeding partners based on animal personality has been studied in various species (e.g., cockatiel *Nymphicus hollandicus* Fox and Millam 2014; Western mosquitofish *Gambusia affinis* Chen et al. 2018; graylag goose *Anser anser* Common et al. 2024), but few studies have specifically explored this compatibility in the context of foraging behavior.

Seabirds, with their diverse foraging strategies and strong pair bonds, offer an excellent model for testing these questions. Behavioral compatibility and coordination within breeding partners are even more important when individuals are constrained by environmental conditions and resource availability (Gabriel and Black 2012) as is the case with marine systems where resources fluctuate in time and space on multiple scales (Durant et al. 2004). Foraging strategy (how, when and where an individual accesses food; Pyke, Pulliam, and Charnov 1977; Martin 1983) can strongly influence reproductive success (Patrick and Weimerskirch 2014; Cohen et al. 2014; Kowalczyk et al. 2015), and coordination of foraging efforts between partners could also affect reproductive success (Black 2001). For example, in the black‐legged kittiwake ( *Rissa tridactyla* ), individuals with less variation in foraging strategy were more likely to fledge a chick (Schlener et al. 2024). In the little auk ( *Alle alle* ), the energy density of food loads delivered to chicks was associated with the level of parental coordination (e.g., trip duration; Grissot et al. 2019). Foraging coordination can enhance chick provisioning, thereby increasing the likelihood of fledgling success (Rishworth and Pistorius 2015; Wojczulanis‐Jakubas, Araya‐Salas, and Jakubas 2018; Piña‐Ortiz et al. 2024).

Understanding how foraging strategies influence reproductive success leads to the question of how morphological traits may affect foraging behavior and, consequently, pairing dynamics. Indeed, individuals could mate with a partner that shares similar morphological measurements, as is the case in few species (e.g., Magellanic penguin *Spheniscus magellanicus* Forero et al. 2001; black‐legged kittiwake Helfenstein, Danchin, and Wagner 2004; little auk Wojczulanis‐Jakubas et al. 2018). Those morphological similarities or differences could be expected to influence behavior in comparison to nonmated individuals; for example, larger individuals can dive deeper and individuals with longer wings may have lower flight costs (e.g., Bowlin and Wikelski 2008; Elliott et al. 2013; Orben et al. 2015; Paredes et al. 2015). Similar morphological features may also lead to partners exhibiting similar behavior. For example, Scopoli's shearwater *Caleonectric diomedea* partners adopted a positive size‐assortative mating by tarsus length, and tarsus length was positively correlated with the duration of incubation shifts (Visalli et al. 2023).

Foraging strategies may differ between breeding partners, as individuals of many seabird species are highly specialized in the selection of prey items, consistently forage in the same areas, and/or show sex‐stereotyped foraging behavior (Woo et al. 2008; Elliott, Gaston, and Crump 2010; Phillips et al. 2017; Courbin et al. 2018; Borrmann et al. 2019; Gulka and Davoren 2019). Given the variability in foraging strategy and the necessity of nest attendance, each mate thus has to adjust its foraging effort and time allocated to the needs of its offspring, while also considering the needs of its partner and itself (Kavelaars et al. 2021). Therefore, independently of their own foraging strategy, breeding partners should coordinate their foraging behavior to increase their reproductive success (Shoji et al. 2015; Bebbington and Hatchwell 2016; Wojczulanis‐Jakubas, Araya‐Salas, and Jakubas 2018; Grissot et al. 2019). For example, pair members of the lesser black‐backed gull ( *Larus fuscus* ) are more similar to each other than to other individuals of the population in both time investment (duration of trips, time spent foraging) and effort (distance traveled, time spent flying; Kavelaars et al. 2021).

Within a breeding pair, having complementary foraging strategies could lead to risk partitioning, where one member of the pair chooses a high‐risk, high‐reward strategy and the other a low‐risk, low‐reward strategy, which could, for example, maximize food delivered to offspring across variable foraging conditions (Elliott, Gaston, and Crump 2010). To explore this further, we focused on thick‐billed murres ( *Uria lomvia* ), which are long‐lived monomorphic and socially monogamous seabirds that show high individual specialization and consistency (daily, weekly and annually) in their foraging strategies (Woo et al. 2008). They are single‐prey loaders (Gaston and Nettleship 1981) that experience high flight costs (Elliott et al. 2013). During breeding, one sex of thick‐billed murre is primarily nocturnal while the other is primarily diurnal, although this relationship can vary among colonies (Gaston and Bradstreet 1993). They have low divorce, high adult philopatry to a breeding site, and obligate biparental care (Paredes, Jones, and Boness 2005; Elliott, Gaston, and Crump 2010; Gousy‐Leblanc et al. 2023), which make them an excellent model for testing whether breeding partners exhibit complementarity in foraging strategies.

We investigated complementarity in breeding pairs in thick‐billed murre from an Arctic colony, where males target shallow‐water benthic (e.g., *Stichaeus* shannies) and invertebrate prey at night, making them risk‐averse compared to females, which target deep‐water benthic (e.g., *Triglops* sculpins) and pelagic prey during the day (Elliott, Gaston, and Crump 2010). If breeding partners exhibit complementarity in foraging strategies, then we expect that partners will be more different in their foraging metrics and foraging areas compared to randomized pairs. We also tested if breeding partners exhibit similarity or dissimilarity in their morphometrics compared to randomized pairs. We expected no difference between mated and nonmated murres.

## 2 Methods

### 2.1 Study Site

We conducted fieldwork at the Coats Island thick‐billed murre colony in Nunavut, Canada (West colony Lat: −82.019, Long: 62.948) during the 2022 and 2023 breeding seasons. This cliff colony hosts approximately 30,000 breeding pairs (Gaston 2002). A long‐term monitoring program studying demography, foraging ecology, and diet of thick‐billed murres has been ongoing since 1981 (Patterson et al. 2024).

### 2.2 GPS Accelerometer Deployments

We captured 108 different adult thick‐billed murres at their breeding sites using a noose pole (*n* = 53 in 2022; *n* = 55 in 2023) during chick‐rearing (July 23 to Aug 2 in 2022; July 20–28 in 2023; with chicks between 2 days and 10 days old). We captured individuals when murres frequently switched with their mate (from 7 am to 11 am) to ensure we had both males and females from different pairs deployed with GPS at the same time. We banded the murres to identify them individually and measured body mass (g). A total of 108 GPS accelerometers were deployed, but only 80 were included in the analyses (7 units were not retrieved, 4 units did not record data and for 17 nests, we were unable to catch the partner). We acknowledge that tags deployment can affect the buoyancy and drag of an individual (Elliott, Davoren, and Gaston 2007), thus we deployed a GPS that is around 1% body mass.

We attached GPS accelerometers (AxyTrek, Technosmart, 9 g) to the murre's back feathers using TESA tape (TESA 4651, Hamburg, Germany). GPS accelerometers were set to record a position fix every 3 min, acceleration at 50 Hz in three axes, and depth at 1 Hz with 0.1 m resolution. We began retrieval 72 h (max 96 h) after deployment. Upon recapture, we measured wing length (mm), head‐bill length (mm), and body mass and removed the GPS accelerometers. Handling time was under 8 min. The breeding partner of each individual was also captured and equipped with a GPS accelerometer the day after the recapture (from 7 pm to 11 pm) using the same method. We did not deploy GPS on the same day on both breeding partners as the impact on parental care would be too high (Paredes, Jones, and Boness 2005; Jacobs, Elliott, and Gaston 2013). During all data collection, we were blind to the sex of each bird. We determined the sex of each bird after data collection via genetics or association with a partner of known sex. We conducted fieldwork in accordance with relevant guidelines and regulations from an Animal Use Protocol approved by the McGill University Animal Care Committee (2015‐7599) and permitted by the Canadian Wildlife Service (Scientific Research Permit SC‐NR‐2022‐NU‐007) and Nunavut government (WL2022‐019; WL2023‐015).

### 2.3 Foraging Metrics

GPS‐accelerometer data were processed in *R* 4.2.2 (R Core Team 2023). We calculated wing‐beat frequency, pitch, diving, step length, and distance from the colony from GPS accelerometer data to define activities (at the colony, diving, flying, or swimming). We used a sampling interval (time step) of 5 min, and estimated missing observations within the trip based on a continuous‐time correlated random walk, using the *crawlWrap* function in the R package *crawl* (McClintock 2017). A full description of the distributions and starting values used for each activity and variable is provided in Table S1. We fitted Hidden Markov Models with the *momentuHMM* package (McClintock and Michelot 2018).

Once activities were classified, we considered murres to be on a foraging trip if they were farther than 1 km away from the colony (to filter locations associated with preening and socializing in the splashdown area adjacent to the colony; Elliott, Woo, and Gaston 2009; Brisson‐Curadeau et al. 2018) and if the trip was longer than 20 min and included at least one dive.

Once we identified foraging trips, for each individual, we calculated foraging and diving metrics for each trip and then averaged each metric throughout the entire deployment. The foraging trip metrics included maximum distance from the colony (km), total distance traveled (km), the mean trip distance (km), trip duration (hours), and mean, maximum, and minimum azimuth (between the colony and the most distant point of the trip). The overall deployment metrics included the average daily distance traveled (km), mean and maximum trip duration (hours), and number of trips per day. We also computed standard deviations for each metric. Diving metrics included maximum dive depth (m), mean dive depth (m), and total dive duration (hrs). To include the time component in the dive depth variable (i.e., controlling diurnal effects on dive depth), we calculated the average depth for each hour of the day. We then calculated for each dive, the difference between individual depth and average depth at the time of the dive. We named this variable differential depth. We calculated maximum and mean differential depth, maximum dive duration (min), mean dive duration (min), the number of dives per day, and standard deviations for each variable. We also calculated for each individual the proportion of time spent in each activity (i.e., at the colony, diving, flying, swimming) during the entire deployment. We used the absolute values in the model.

### 2.4 Foraging Locations

To identify the foraging area for each murre, we conducted a kernel utilization distribution (KUD) using the *KernelUD* function from the *adehabitatHR* package (Calenge 2006). We used all locations within all foraging trips. We calculated kernel smoothing parameters separately for each individual using the *ad hoc* method and we averaged the utilization distributions (UD) across all individuals. We specified a smoothing bandwidth (*h*) of 2720 m (which is the *href* averaged across all individuals) and a grid of 200 m. We obtained the utilization distribution core range of 50% and 95% contour polygons within the home range of each individual.

To investigate if breeding partners forage in the same locations, we estimated the degree of overlap between the core foraging area (50% UD) of an individual and all other individuals of the opposite sex that were recorded in the same year. We used the Bhattacharyya's Affinity (BA) method implemented in the *kerneloverlaphr* function to analyze the degree to which a male and a female forage in the same area (i.e., 0 signifying no overlap in UDs and 1 is identical UDs; Fieberg and Kochanny 2005).

### 2.5 Statistical Analysis

#### 2.5.1 Differences Between Males and Females

We first used a paired t‐test to compare the body mass of individuals before and after GPS deployment. We then applied unpaired t‐tests to compare the average foraging behavior and morphometric variables between male and female thick‐billed murres.

#### 2.5.2 Differences Between Real Breeding Pairs and Randomized Pairs

For all foraging and diving metrics listed and for morphometrics (average body mass, wing length, head‐bill length), we calculated the difference between each female and each male separately per year (female trait‐male trait: 19 females × 19 males =361 potential breeding pairs in 2022; 21 females × 21 males = 441 potential breeding pairs in 2023). We used absolute values of the difference to examine the dissimilarity between paired females and males, as we were interested in the difference between them and not the sex with the higher value. For each potential breeding pair (*n* = 802 [361 + 441]), we added a type of pair variable with a value of 1 for real breeding pairs (*n* = 40) and 0 for the other randomized (not true) breeding pairs (*n* = 762). This way, we compared foraging traits differences of all females with all males available in each year. The type of pair is our response variable in the models. We tested for collinearity with a correlation matrix among all the foraging and diving variables. We only included one variable when variables had *R* > 0.8 in the same model. The correlated variables were mostly the maximum or average of a given metric (e.g., mean dive duration or maximum dive duration), and so we selected the average variable. To facilitate model convergence, we standardized (centered and scaled) the maximum distance traveled, the total distance traveled, the mean trip distance, and the wing length.

To determine whether breeding partners are more similar or dissimilar than randomized pairs in their foraging strategies, we estimated the probability of the type of pairs constituting real breeding pairs (0 or 1) using logistic regression in a generalized linear mixed‐effects model (GLMM). We used the *glmmTMB* function (Brooks et al. 2017), with a binomial distribution and a logit link. We used female and male bird identities as a nested random effect with year. We added a weighted argument to the model to correct for the unbalanced number of randomized pairs versus real pairs (762 values vs. 40 values). We tested several models (*n* = 30) with the differences between males and females in foraging, diving, and morphometrics listed as explanatory variables.

To evaluate if breeding partners overlapped in their core foraging area, we used the same model structure with Bhattacharyya's Affinity overlap index between individuals as an explanatory variable. We transformed the Bhattacharyya's Affinity overlap index using a log(*x* + 1) transformation to allow model convergence. We used a model selection procedure using the Akaike information criterion (AIC) to determine which models best explained the probability of the type of pairs constituting real breeding pairs. We performed all statistical analyses using *R* 4.2.2 (R Core Team 2023).

## 3 Results

### 3.1 Differences Between Males and Females

**FIGURE 1:**
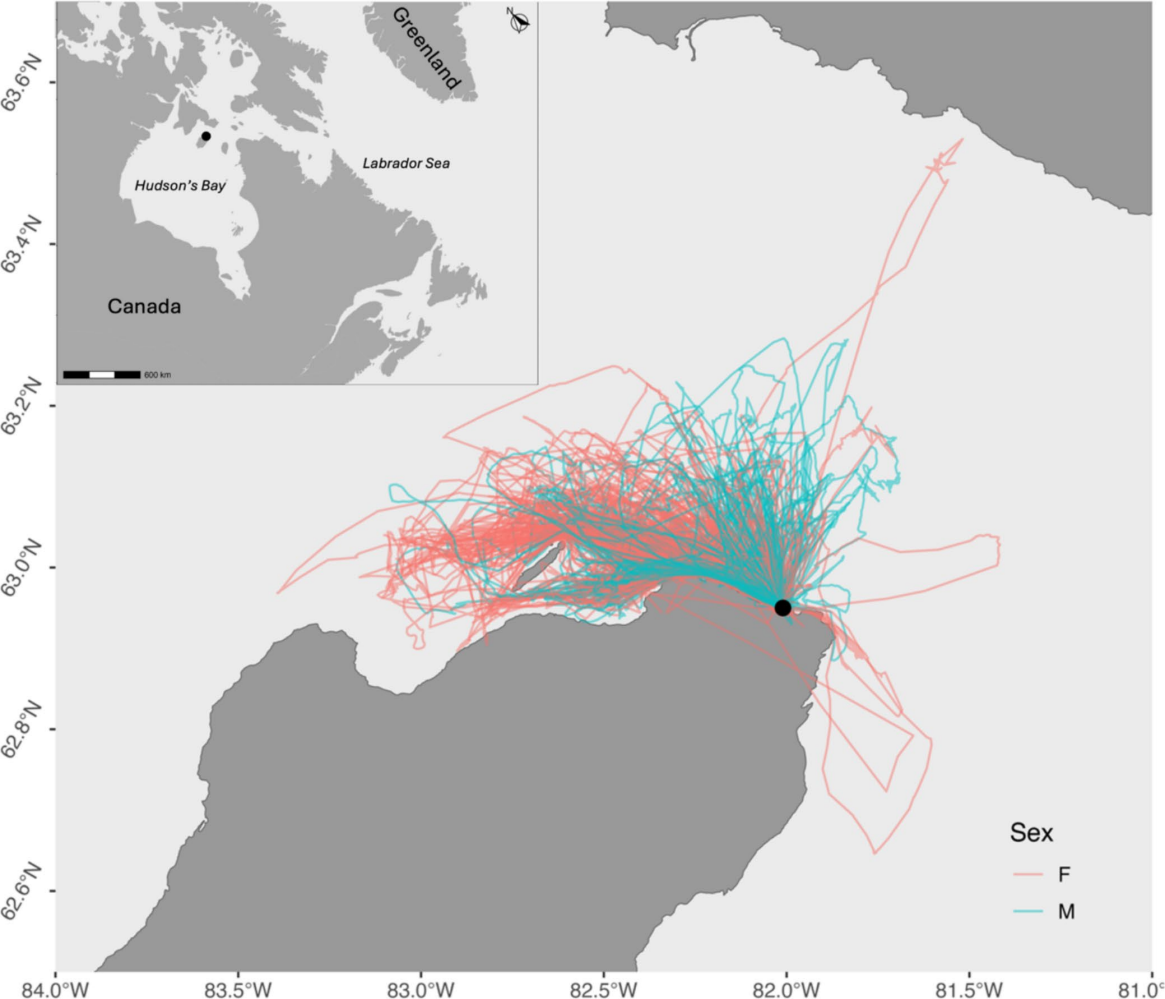
Foraging trips (*n* = 652) of 40 female (pink) and 40 male (blue) thick‐billed murres during the chick‐rearing period at the Coats Island colony (black dot).

We analyzed 652 foraging trips across 40 breeding pairs (19 pairs in 2022; 21 pairs in 2023; Figure 1). Body mass was higher, on average, before (981 ± 65 g) than after the deployment (after 961 ± 61 g; paired *t*‐test: *t* = 5.38, df = 82, *p* < 0.001). Females and males over all samples differed from each other in several foraging metrics including maximum distance from the colony, total, and average distance traveled, and average azimuth (Table 1). The number of dives was significantly higher for females compared to males (Table 1). Female and male thick‐billed murres were similar in their morphometrics (Table 1).

**TABLE 1:** Summary of foraging strategy and morphometrics for thick‐billed murres from Coats Island instrumented with GPS‐ accelerometers during the chick‐rearing period of 2022 and 2023. Data are mean ± standard deviation. *p‐*values are from a Student's *t*‐tests. Values that are statistically significant at *α* < 0.05 are highlighted in bold.

Foraging metrics	Females	Males	*p*
	(*n* = 40, 339 trips)	(*n* = 40, 313 trips)	
**Maximum distance from colony (km)**	41.5 ± 12.9	31.0 ± 9.9	**< 0.001**
**Total distance traveled (km)**	480 ± 136	306 ± 123	**< 0.001**
**Average distance traveled (km)**	64.7 ± 30.3	43.6 ± 21.6	**< 0.001**
Total trip duration (h)	33.6 ± 6.4	31.7 ± 8.0	0.249
Average trip duration (h)	4.5 ± 1.8	4.5 ± 1.7	0.965
Average number of trips	8 ± 3	8 ± 3	0.313
Number of trips daily	6 ± 2	6 ± 2	0.315
Maximum Azimuth (°)	310 ± 26	314 ± 40	0.598
**Average Azimuth (°)**	267 ± 31	242 ± 57	**0.018**

Diving metrics	(*n* = 39, 467 dives)	(*n* = 39, 355 dives)	
Maximum differential depth (m)	−0.63 ± 18.17	−0.23 ± 9.98	0.900
Average differential depth (m)	−0.28 ± 9.1	0.01 ± 6.07	0.871
Total time spent diving (h)	4.1 ± 2.4	3.3 ± 2.3	0.104
Average dive time (min)	21.3 ± 9.2	21.7 ± 10.3	0.876
**Number of dives**	12 ± 5	9 ± 5	**0.032**

Morphometric measurements	(*n* = 37)	(*n* = 38)	
Body mass (g)	970 ± 60	972 ± 64	0.781
Wing length (mm)	219 ± 6	221 ± 6	0.135
Head‐bill length (mm)	101 ± 3	102 ± 3	0.245

### 3.2 Differences Between Real Breeding Pairs and Randomized Pairs

**FIGURE 2:**
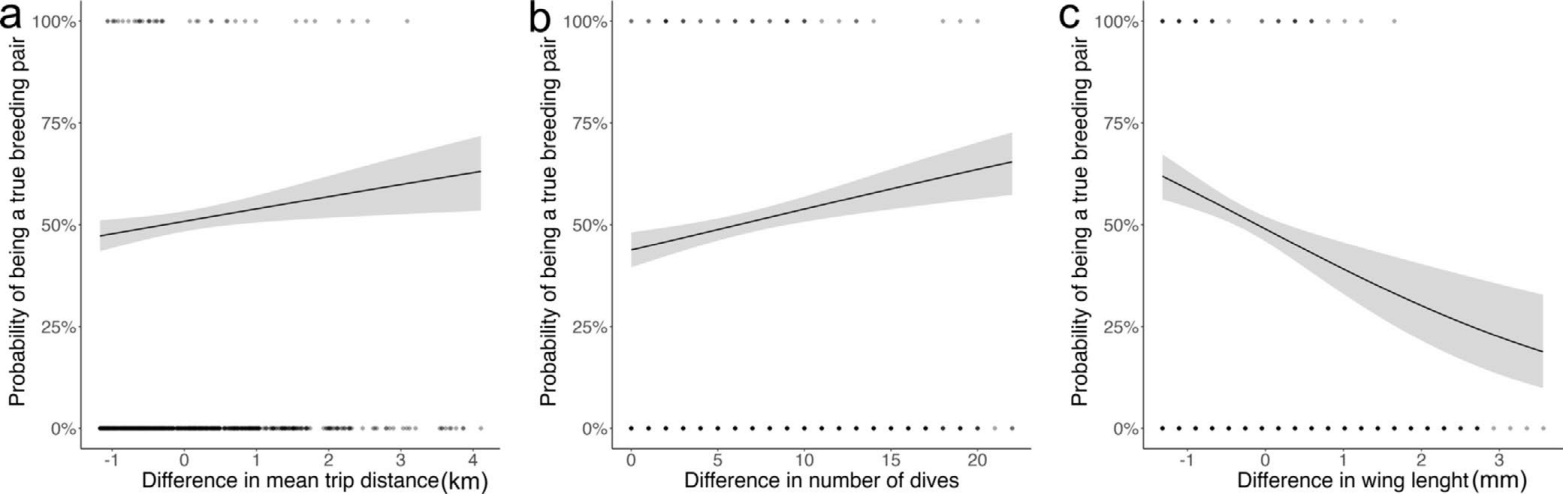
Probability of being a true breeding pair versus randomized pair as a function of difference between a female and a male in (a) mean foraging trip distance, (b) number of dives during a foraging trip, and (c) wing for thick‐billed murres at Coats Island during chick rearing.

The best model selected for foraging metrics included the difference in mean trip distance between partners (Table 2a). The difference in mean trip distance was higher for real breeding pairs compared to randomized pairs (estimate = 0.12, intercept = 0.03; *p* = 0.01; 95% CI = 0.027–0.217; Figure 2a). For the diving metrics, the selected model included the difference in number of dives (Table 2b). The difference in number of dives was also higher for real breeding pairs compared to randomized pairs (estimate = 0.04 intercept = −0.25; *p* = 0.0002; 95% CI = 0.019–0.061; Figure 2b).

**TABLE 2:** Model selection based on AICc for the probability of being a real breeding pair based on (a) foraging metrics, (b) diving metrics, (c) morphology and (d) the Bhattacharyya's Affinity overlap (BA) during foraging trips in thick‐billed murres at Coats Island during chick‐rearing. Pair: Type of breeding pair (1 = real, 0 = randomized pairs).

Models	AICc	ΔAICc	AICcweight	Deviance
(a)				
Pair ~ Mean_dist_trip + (1|Band_F/Year) + (1|Band_M/Year)	2178.76	0.00	0.43	2166
Pair ~ SdTime + (1|Band_F/Year) + (1|Band_M/Year)	2181.42	2.66	0.11	2169
Pair ~ SdDiving + (1|Band_F/Year) + (1|Band_M/Year)	2181.67	2.91	0.10	2169
Pair ~ Max_dist_trip +(1|Band_F/Year) + (1|Band_M/Year)	2181.89	3.13	0.09	2169
Pair ~1 + (1|Band_F/Year) + (1|Band_M/Year)	2183.13	4.37	0.05	2173
Pair ~ SdMaxdist + (1|Band_F/Year) + (1|Band_M/Year)	2184.02	5.26	0.03	2171
Pair ~ Total_trip_duration + (1|Band_F/Year) + (1|Band_M/Year)	2184.38	5.62	0.02	2172
Pair ~ MeanAzimuth + (1|Band_F/Year) + (1|Band_M/Year)	2184.54	5.78	0.02	2172
Pair ~ SdSwim + (1|Band_F/Year) + (1|Band_M/Year)	2184.61	5.85	0.02	2172
Pair ~ SdFly + (1|Band_F/Year) + (1|Band_M/Year)	2184.68	5.91	0.02	2172
Pair ~ Total_dist_traveled + (1|Band_F/Year) + (1|Band_M/Year)	2184.71	5.95	0.02	2172
Pair ~nbTrip + (1|Band_F/Year) + (1|Band_M/Year)	2184.79	6.03	0.02	2172
Pair ~ ndTrip/day + (1|Band_F/Year) + (1|Band_M/Year)	2185.14	6.38	0.02	2173
Pair ~ SdDist + (1|Band_F/Year) + (1|Band_M/Year)	2185.16	6.40	0.02	2173
Pair ~ SdAz + (1|Band_F/Year) + (1|Band_M/Year)	2185.16	6.40	0.02	2173
(b)				
Pair ~ nbdives + (1|Band_F/Year) + (1|Band_M/Year)	2031.45	0.00	0.92	2019
Pair ~ Duration + (1|Band_F/Year) + (1|Band_M/Year)	2037.71	6.27	0.04	2025
Pair ~ SdDiff_Max_Depth + (1|Band_F/Year) + (1|Band_M/Year)	2039.43	7.99	0.02	2027
Pair ~ Sd_DiveDuration + (1|Band_F/Year) + (1|Band_M/Year)	2039.94	8.50	0.01	2027
Pair ~1 + (1|Band_F/Year) + (1|Band_M/Year)	2043.62	12.17	0.00	2029
Pair ~ Mean_dive_duration + (1|Band_F/Year) + (1|Band_M/Year)	2044.68	13.23	0.00	2032
Pair ~1 + (1|Band_F/Year) + (1|Band_M/Year)	2045.15	13.70	0.00	2033
Pair ~ SdMeanDepth + (1|Band_F/Year) + (1|Band_M/Year)	2045.64	13.93	0.00	2034
Pair ~ Diff_mean_depth + (1|Band_F/Year) + (1|Band_M/Year)	2045.64	14.19	0.00	2034
(c)				
Pair ~ Diff_wing + (1|Band_F/Year) + (1|Band_M/Year)	1843.88	0.00	1.00	1828
Pair ~1 + (1|Band_F/Year) + (1|Band_M/Year)	1885.58	41.70	0.00	1873
Pair ~ Diff_headbill + (1|Band_F/Year) + (1|Band_M/Year)	1886.00	42.12	0.00	1875
Pair ~ Diff_mass + (1|Band_F/Year) + (1|Band_M/Year)	1887.00	43.72	0.00	1875
(d)				
Pair ~ 1 + (1|Band_F/Year) + (1|Band_M/Year)	1477.06	0.00	0.73	1042
Pair ~ BA + (1|Band_F/Year) + (1|Band_M/Year)	1477.04	1.98	0.27	1042

**FIGURE 3:**
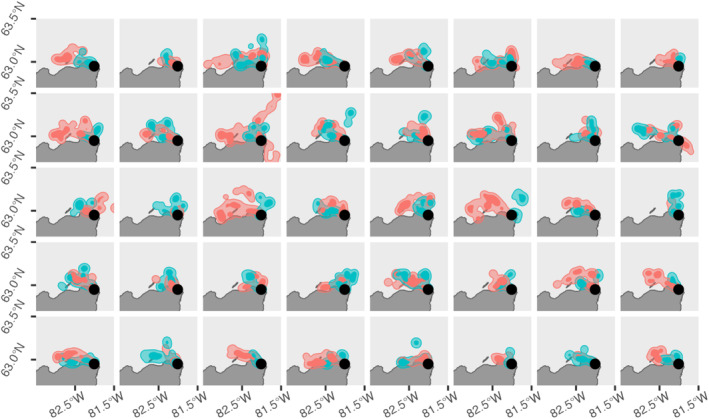
Foraging areas used by female (pink) and male (blue) thick‐billed murres from 40 breeding pairs at Coats Island during the 2022 and 2023 chick‐rearing period. The core foraging area (50% kernel density estimate) is indicated by darker contours and the broad foraging range (95% kernel density estimate) is indicated by lighter contours. Smoothing parameter = 2720 m and grid = 200 m.

We found no effect of kernel overlap of the foraging core area on the probability of being a true breeding pair. The best model was the null model (Table 1d). True breeding pairs did not overlap more than randomized pairs (Figure 3). The selected model included the difference in wing length (Table 2c). The difference in wing length was smaller in real breeding pairs compared to randomized pairs (estimate = −0.34 intercept = −0.04; *p* < 0.001; 95% CI = −0.59, −0.21; Figure 2c).

## 4 Discussion

In this study, we found that thick‐billed murre breeding partners at Coats Island have dissimilar foraging strategies during chick‐rearing, showing complementarity in foraging behavior within breeding partners. Specifically, breeding partners had different foraging trip distances and numbers of dives. Thus, birds with short trips and few dives tended to be paired with individuals with long trips and many dives. Despite being monomorphic, individuals were paired more frequently with a partner of similar wing length.

These results align with the conceptual model proposed by Elliott, Gaston, and Crump (2010), where data from a combination of time‐depth‐temperature recorders, observations from chick feeding, and stable isotopes revealed that breeding partners with different foraging strategies (one risk‐averse with one risk‐tolerant) have a higher chance of their offspring surviving compared with partners with similar foraging strategies. Differences in foraging strategies within pairs is probably related to both individual variation and sex‐specific variation and could in part explain the diversity in foraging strategies in this species. Indeed, if variation in foraging metrics was only related to a male–female difference, we would not have found a significant difference of some foraging metrics between real and randomized breeding pairs. Foraging strategy is highly variable for both sexes at this colony (Woo et al. 2008; Elliott, Woo, and Gaston 2009). Regardless of sex or age, the main component of variation in foraging behavior was between individuals (i.e., individual repeatability over a week: average dive depth = 0.95, average flight time = 0.56, dive shape 0.95; Woo et al. 2008). Variation in foraging behavior and chick diet occurs largely because individual murres specialize on a single foraging strategy regardless of whether they are provisioning their chick or themselves (Woo et al. 2008). The sex‐specific difference in foraging strategy is most pronounced during chick‐rearing, and perhaps most important near the end of the period (Elliott, Gaston, and Crump 2010). Indeed, only the male stays with the flightless chick after colony departure. Once at sea, males need to stay in contact with their chick and this may be improved by risk‐averse foraging (e.g., taking invertebrates or shallow‐water benthic prey which do not need long or deep dives; Elliott et al. 2009; Elliott, Gaston, and Crump 2010). Notably, this sex‐specific foraging strategy is not present in all thick‐billed murres colonies. Indeed, in Greenland, male and female thick‐billed murres showed no differences in foraging distribution, foraging habitat or diet (Huffeldt et al. 2021).

Differences in foraging strategy and diet within breeding pairs could explain how parental duties are divided and whether compensation occurs within pairs in prey types consumed and delivered to chicks (Harris et al. 2016). One breeding partner apparently chooses a high‐risk, high‐reward strategy (i.e., high number of dives or foraging farther away, likely leading to provisioning offspring with large schooling fish) and the other partner adopts a low‐risk, low‐reward strategy (i.e., low number of dives and foraging closer to the colony, likely leading to provisioning offspring with invertebrates or small fish) which collectively, appears to maximize chick growth rates (Elliott, Gaston, and Crump 2010). As central‐place foragers, murres create a zone of prey depletion around the colony (“Storer‐Ashmole's halo”; Elliott et al. 2009; Patterson et al. 2022). If one partner forages closer to the colony and the other forages farther away, they could target different types of prey allowing a higher probability of bringing food to the chick. Nonetheless, dissimilar foraging strategies within breeding pairs contrasts with several other seabirds. In Kerguelen shags ( *Phalacrocorax verrucosus* ), mates followed similar bearings, overlapped in foraging areas, and foraged at similar trophic levels (Camprasse et al. 2017). In chick‐rearing lesser‐backed gulls, mates were more similar to each other than to other individuals in their foraging trip duration, distance traveled and time spent flying or foraging (Kavelaars et al. 2021).

Our finding of complementary foraging behavior in thick‐billed murre breeding partners suggests that, despite being monomorphic, individuals exhibit different foraging strategies compared to their partners, regardless of sex. The behavioral compatibility of breeding partners in their foraging strategies may enable them to provide more effective care (e.g., provisioning) to their chick, as has been established for other behavioral traits (e.g., animal personality; Both et al. 2005; Spoon, Millam, and Owings 2007; Schuett, Dall, and Royle 2011; Collins et al. 2019). This study is one of the few to investigate the foraging behavior of breeding partners and could serve as a starting point to assess whether complementarity in foraging strategy is common in seabird breeding pairs.

Whether individuals chose partners with a different foraging strategy, or foraging strategies of breeding partners diverged after pairing is unclear. Thick‐billed murres are long‐lived (> 20 years) and faithful to their partners and on Coats Island, younger, less experienced birds are not as successful in fledging their chick as older, experienced pairs (De Forest and Gaston 1996). Partners may be coordinating or changing their foraging strategies with time to increase chick growth rates. Regardless, complementarity of foraging strategy is clearly a key characteristic of murre breeding pairs. Future studies could evaluate if the breeding partners change their foraging strategy after pairing to coordinate with their mate or if individuals actively choose their breeding partner based on complementarity of their foraging strategies. Future studies also could compare foraging strategies of murres during the pre‐laying and incubation periods to assess if the same pattern of disassortative mating occurs then. It is possible that the potential advantages of having different foraging strategies are only detectable during chick‐rearing, when energy demands increase as adults forage to meet their own demands and those of the chick (Montevecchi, Birt‐Friesen, and Cairns 1992; Welcker et al. 2015). Having those studies will help understand the mechanism (active choice vs. coordination) behind the differences in feeding strategies that we detected here (Jiang, Bolnick, and Kirkpatrick 2013; Dingemanse, Class, and Holtmann 2021; Munson et al. 2021).

### 4.1 Potential Impacts on Fitness

Complementary and different foraging strategies between breeding partners could favor better reproductive success for pairs showing this compatibility. Indeed in other seabird species, differences in foraging strategies within pairs is related to higher reproductive success (Watanuki 1992). In our study, we could not determine if pairs with different foraging strategies had higher reproductive success. Fledging success is the only reproductive success proxy available in our system, and most breeding pairs that we tracked had successful fledging (34/38 pairs). We did not have enough statistical power to test if different foraging metrics within the pair (number of dives and average foraging trip distance) influenced fledging success. In alcids, postfledging survival may be more important than fledging success; adequate food and energy at the colony may be critical for chicks to survive postfledging (Harris, Frederiksen, and Wanless 2007; Morrison et al. 2009; Elliott et al. 2017). Indeed, a direct link between fledging mass and postfledging survival and recruitment has been found in many other species (Maness and Anderson 2013), such as wedge‐tailed shearwaters *Ardenna pacifica* (Swanson et al. 2023).

### 4.2 Breeding Partners' Morphology

Thick‐billed murres at our colony paired with partners of similar wing length compared to randomized pairs. In thick‐billed murres, body mass and size can be positively associated with deep diving and negatively associated with long flights, suggesting that morphology influences foraging and commuting efficiency in this species (Paredes et al. 2015). Because no associations occurred in other parameters more closely tied to “size” (i.e., head‐bill length or body mass), similar wing length is likely associated with flight parameters. Two general hypotheses have been proposed to explain assortative mating: (1) assortative mating could be an adaptative response to direct or indirect selective pressures that can impact the fitness of individuals (or their offspring), or (2) it can be an incidental consequence of other constraints that cause individuals with similar attributes to mate (Jiang, Bolnick, and Kirkpatrick 2013). We argue that the latter is applicable in our system. For example, members of a pair may have similar wing lengths due similarity in age (assortative mating by age is common in seabirds; Jouventin, Lequette, and Dobson 1999, Ferrer and Penteriani 2003, Ludwig and Becker 2008) or breeding site (i.e., whether the site tends to abrade feathers or not), perhaps because high quality individuals select other high quality individuals or nest sites.

Alternatively, migration distance and/or timing of arrival at the colony could lead individuals to mate with a partner of similar wing length, as birds with longer wings create more lift and have lower flight costs (Elliott et al. 2013). Indeed, several bird species show a relationship between wing length and migration distance (e.g., Marchetti, Price, and Richman 1995; Nowakowski, Szulc, and Remisiewicz 2014; Ożarowska, Zaniewicz, and Meissner 2021; Matyjasiak et al. 2023). We speculate that individuals with longer wings could mate with individuals with longer wings if they arrive at the same time postmigration, as they would encounter each other at the same time during the prebreeding season. A future study could assess migration distance, nonbreeding locations, and timing of arrival of breeding partners to try to understand the mechanism behind assortative mating for wing length. Breeding pairs of thick‐billed murres in Greenland showed spatial separation but similar photic environment during winter (Huffeldt et al. 2024). It will be interesting to test for the same pattern at Coats Island. Assortative mating based on wing length was also found in little auks, and the authors also suggested that this could be linked to their migration patterns (Wojczulanis‐Jakubas et al. 2018).

### 4.3 Tag Effect

Although we found that the body mass of the individuals decreased after the 3 days deployments of tags, in thick‐billed murres, body mass of adult decreases as the chick rearing period advances (Croll, Gaston, and Noble 1991). We acknowledge that the tag could be affecting the foraging behavior of individuals (e.g., Evans et al. 2020), but we tried to mitigate the effects by using a small tag (1% body mass).

### 4.4 Summary

Overall, this study is one of few comparisons of foraging strategies between breeding partners during chick‐rearing in seabirds. Despite being similar physically, thick‐billed murres demonstrate different foraging strategies within breeding pairs. Breeding partners of thick‐billed murres at Coats Island had different trip distances and numbers of dives, but similar wing lengths compared to randomized pairs of birds. Several selection pressures may influence the traits of partners, including active mate choice, cooperation and coincidence of timing and location of migration or nonbreeding. Investigating the timing of formation of the pair bond could help in understanding mate pairing in the species. In thick‐billed murres, diet, foraging strategies and sex‐specific foraging behavior vary even between nearby colonies (with similarities among very distant colonies) implying that such patterns are context‐rather than species‐specific (Elliott, Gaston, and Crump 2010; Huffeldt et al. 2021). Evaluating if differences in foraging strategies between breeding partners is colony‐specific would be interesting. The balance between sex‐specific foraging strategies and complementarity in foraging strategies between breeding partners could contribute to the diversity of individual foraging strategies present in this species.

## Author Contributions

**Marianne Gousy‐Leblanc:** conceptualization (equal), data curation (lead), formal analysis (lead), investigation (lead), methodology (lead), visualization (lead), writing – original draft (lead), writing – review and editing (lead). **Allison Patterson:** data curation (supporting), formal analysis (supporting), methodology (supporting), visualization (supporting), writing – review and editing (supporting). **H. Grant Gilchrist:** funding acquisition (equal), resources (equal), writing – review and editing (supporting). **Vicki L. Friesen:** conceptualization (lead), resources (supporting), supervision (equal), writing – review and editing (supporting). **Kyle H. Elliott:** conceptualization (lead), funding acquisition (equal), resources (equal), supervision (equal), writing – review and editing (supporting).

## Ethics Statement

Research activities on Coats Island, NU, were in accordance with relevant guidelines and regulations from an Animal Use Protocol approved by the McGill University Animal Care Committee (2015‐7599) and permitted by the Canadian Wildlife Service (Scientific Research Permit SC‐NR‐2022‐NU‐007) and Nunavut government (WL2022‐019; WL2023‐015).

## Conflicts of Interest

The authors declare no conflicts of interest.

## Supporting information


**Table S1.** Starting values for the state‐dependent probability distribution parameters for variables used in the hidden Markov model to classify four activities of thick‐billed murres.

## Data Availability

The data and code are available on Figshare: https://figshare.com/s/e856b63449d0d12a38ea.
