# Supplementary material for: Breeding Partners Have Dissimilar Foraging Strategies in a Long‐Lived Arctic Seabird
Source: Ecol Evol. 2025 Jan 23;15(1):e70816. doi: 10.1002/ece3.70816 (PMC11755065; doi:10.1002/ece3.70816)
Supplement: Supplementary file 1 — Table S1. Starting values for the state‐dependent probability distribution parameters for variables used in the hidden Markov model to classify four activities of thick‐billed murres. [file ECE3-15-e70816-s001.docx]

**SUPPLEMENTARY MATERIAL**

**Table S1:** Starting values for the state‐dependent probability distribution parameters for variables used in the hidden Markov model to classify four activities of thick‐billed murres. SD= standard deviation; WBF= wing beat frequency.

|  |  |  |  |  |  |  |
| --- | --- | --- | --- | --- | --- | --- |
| **Variable** | **Family** | **Parameter** | **Flying** | **Diving** | **Swimming** | **Colony** |
| WBF | Gamma | Mean | 8 | 1 | 1 | 1 |
|  |  | SD | 1 | 1 | 1 | 1 |
| Diving | Bernoulli | Probability | 0.00001 | 0.999999 | 0.00001 | 0.00001 |
|  |  |  |  |  |  |  |
| Off colony | Bernoulli | Probability | 0.9 | 0.9 | 0.9 | 1E-50 |
|  |  |  |  |  |  |  |
| Pitch | Normal | Mean | 0 | 0 | -5 | 45 |
|  |  | SD | 10 | 50 | 5 | 25 |
| Step length | Gamma | Mean | 500 | 50 | 50 | 10 |
|  |  | SD | 500 | 50 | 50 | 10 |
